# Diagnostic value of computed tomography and magnetic resonance imaging in ovarian malignant mesothelioma

**DOI:** 10.1186/s12880-023-01165-5

**Published:** 2023-12-14

**Authors:** Hai-tao Wang, Ai-jing Li, Ji-yong Gao, Liang-jiong Wang, Yu-tao Wang, Wen-ying Yu, Jian Zhang

**Affiliations:** 1 Department of radiology, The First Affiliated Hospital of Ningbo University, No.247 Renmin Road,Jiangbei District, Ningbo, 315020 China; 2 Department of radiology, Hwa Mei Hospital,University of Chinese Academy of Scienecs, Ningbo, 315016 China; 3 Department of radiology, Ningbo Women & Children’s Hospital, Ningbo, 315031 China; 4 Department of radiology, Ningbo Medical Center Lihuili Hospital, Ningbo, 315042 China; 5 Department of Pathology, Ningbo Clinical Pathology Diagnosis Center, No.685 Huanchengbei Road,Jiangbei District, Ningbo, 315021 China; 6 Shanghai University, Shanghai Universal Medical Imaging Diagnostic Center, Building 8, 406 Guilin Road, Xuhui District, Shanghai, 201103 China

**Keywords:** Diagnosis, Magnetic resonance imaging, Mesothelioma, Ovarian tumor, Treatment, X-ray computer

## Abstract

**Objective:**

To investigate the diagnostic value of computed tomography (CT) and magnetic resonance imaging (MRI) in ovarian malignant mesothelioma (OMM).

**Methods:**

The clinical and imaging data of 10 pathologically-confirmed OMM patients were analyzed retrospectively.

**Result:**

(1) The patients were 27 years to 70 years old, with an average age of 57.2 ± 15.4 years. Seven patients reported abdominal distension and pain, 1 reported lower abdominal discomfort and decreased appetite, and 2 patients had no symptoms. (2) Two cases of localized OMM with incomplete semi-annular “capsule” observed around the localized OMM tumors were reported while 8 cases had diffuse OMM in which the tumor parenchyma showed isointense or slightly hypointense on T1WI, inhomogeneous hyperintense on T2WI, and obviously hyperintense on DWI, with obvious inhomogeneous enhancement after enhancement. Diffuse OMM was not mainly composed of ovarian masses and was mainly characterized by mild ovarian enlargement, nodular and irregular thickening of the peritoneum, cloudy omentum, unclear fat gap, and reticular or irregular thickening, which can fuse into a “cake-shape”. (3) All 10 patients underwent surgery, while 9 patients underwent systemic chemotherapy or immunotherapy after surgery. All patients with localized OMM survived. Out of the 8 diffuse-type patients, 5 died, 1 was lost to follow-up, and 2 survived.

**Conclusion:**

OMM has certain clinical and imaging characteristics. There is no liquefaction, calcification, or partition in the tumor. The ovarian enlargement in the diffuse lesion is not significant. The diffuse thickening of the peritoneum and omentum with early appearance of mural nodules and ascites in the upper abdomen, help the diagnosis of OMM.

Malignant mesothelioma (MM) affects mesothelial cells and is a highly-malignant type of cancer. It can affect the pleura, peritoneum, pericardium, testicular tunica vaginalis, and ovary, [1] among which the pleura is the most affected, while the peritoneum is rarely affected, especially in the ovaries [2]. The extremely low incidence of ovarian malignant mesothelioma (OMM) and the lack of specific clinical manifestations and laboratory indicators often leads to misdiagnosis especially as diagnosis is mainly dependent on the pathology. Previous imaging studies on OMM mainly focused on case reports, which described the unilateral imaging features. In this paper, the computed tomography (CT) and magnetic resonance imaging (MRI) features of 10 OMM cases were retrospectively analyzed. Backed by relevant literature, the diagnostic values of CT and MRI are discussed to improve the diagnosis of OMM.

## Materials and methods

### General information

The clinical, laboratory, imaging, and pathological data of 10 female patients with pathologically-confirmed OMM between September 2013 and July 2022 were collected. The patients were 27 years to 70 years old, with an average age of 57.2 ± 15.4 years. This study was approved by the Ethics Committee of our hospital.

### Inclusion and exclusion criteria

Inclusion criteria: (1) patients with OMM, confirmed by pathology tests; (2) Patients with pre-operative CT enhancement or MRI enhancement scanning; (3) Patients in whom invasive examinations such as puncture biopsy and tumor-related treatment have not been performed before image examination and operation; (4) Patients whose clinical and pathological data are complete.

Exclusion criteria: (1) Patients with previous medical history of other malignant tumors; (2) Patients with OMM recurrence (3) Poor image quality of scans, which affects detailed evaluation.

### Examination methods

Spiral CT with more than 16 rows was adopted for CT examination. The scanning range started from the diaphragmatic surface to the lower margin of pubic symphysis. The four-stage scans included plain scan, arterial (25 ~ 30) s, portal (60 ~ 65) s, and delayed (110 ~ 120) s. Scanning parameters: tube voltage 120 kV, milliampere second 150-210mAs, ball tube rotation time 0.5s/cycle, detector collimation width 100 × 0.5 mm, data reconstruction thin layer thickness 1 mm, layer spacing 0.8 mm, field of view 350 mm × 350 mm, radiation dose 46-65.2mGy. Contrast agent injection procedure: A double-barrel, high-pressure syringe was used to inject 100 mL of non-ionic contrast agent, iodophor (370 mg I/mL), at a rate of 4.5 ~ 5.0 mL/s, followed by 20 mL of 0.9% normal saline.

The Siemens 1.5T superconducting MRI imaging system scanner was used for MRI scanning. The body coil was used for scanning. The scanning range included the whole pelvic cavity, mainly in transverse and coronal position, supplemented by sagittal position. Routine scanning sequence included T1WI, T2WI or fat suppression T2WI, DWI (b value 600–1000), and enhancement scanning sequence inclulded T1WI or fat suppression T1WI. Spin echo sequence (TSE FS T2WI TR > 2500ms, TE 80-100ms, layer thickness 4–6 mm, interval 1-1.5 mm, SE T1WI TR 300-500ms, TE 10-20ms) and gradient echo sequence (T1WI TR 100-200ms, TE < 15ms, FL50-80) were used. The dose for Gd-DTPA enhanced scan was 0.1mmol/kg.

### Image analysis

The images were independently reviewed by two radiologists with more than 10 years of work experience. If there was disagreement between them, another radiologist with more than 10 years of work experience was invited to discuss pertinent issues. The three had to agree upon any issue after consultation. Imaging examination and factors for observation: tumor location, size, cystic and solid nature, calcification, range, peritoneal changes, omental changes, lymph node metastasis, vascular invasion, and strengthening characteristics.

### Pathological examination

All the specimens were fixed in 10% neutral formalin and were subjected to routine adequate sampling, paraffin embedding, HE staining, observation under light microscope, and immunohistochemical staining. The two-step method of En Vision was used for immunohistochemical staining. The antibodies used included CK(pan), CK7, EMA, PAX8, Calretinin, D2-40, CK5/6, WT-1, MC(HBME1), EA(Ber-EP4), MOC31, CD15, ER, and PR. They were all purchased from Roche Diagnostics (Shanghai) Co., Ltd., Fuzhou Maixin Co., Ltd., and Beijing Zhongshan Jinqiao Co., Ltd. Each operation was strictly in accordance with the instructions mentioned in the product catalog. The diagnosis was jointly made by two experienced pathologists.

### Treatment and follow-up

According to the imaging characteristics of the tumor and the patient’s condition, follow-up was conducted either via telephone or as outpatient. Follow-ups were conducted once every two months within six months post operation and once every three months subsequently, to assess prognosis as well as to check for tumor recurrence, metastasis, and survival status until the patients were lost to follow-up or died. The deadline for follow-up was October 2022.

## Results

### General clinical data

Among the 10 OMM cases, 7 had abdominal distension and pain, 1 had lower abdominal discomfort and decreased appetite, and 2 cases had no symptoms. The CA125 levels in all the 10 patients were slightly or moderately elevated, and the average preoperative CA125 level was 93.24±80.89 U/mL (Table 1). CA153 was slightly elevated in 7 patients, and CA199 and other tumor markers were normal. One patient had a history of asbestos exposure, while the etiology of the remaining patients was unknown.

**Table 1:** General information of patients

**No.**	**Age (in years)**	**Symptom**	**CA125(U/ml)**	**CA153(U/ml)**
1	36	No symptom	23.9	11.6
2	47	Abdominal distension, Abdominalgia	92.7	7.7
3	56	Abdominal distension, Abdominalgia	111.3	5.8
4	68	Lower abdominal discomfort, decreased appetite	219.4	22.9
5	67	Abdominal distension, Abdominalgia	247.4	81.5
6	70	Abdominal distension, Abdominalgia	25.4	17.5
7	69	Abdominal distension, Abdominalgia	94.3	32.2
8	65	Abdominal distension, Abdominalgia	56.8	80.8
9	67	Abdominal distension, Abdominalgia	42.8	23.7
10	27	No symptom	18.4	19.5

### Imaging examination results

Six patients underwent contrast-enhanced CT of the whole abdomen, while four underwent contrast-enhanced MRI of the pelvis. In this study, according to the classification method of peritoneal malignant mesothelioma, OMM was divided into limited type and diffuse type based on tumor scope and imaging manifestations. The tumors that are localized in the ovary or with local invasion are classified as localized tumors and the tumors that have metastasized to the peritoneum, omentum, and mesenterium, or that have multiple centers are called diffuse tumors. In this group, there were two cases of localized type and eight cases of diffuse type (Table 2) tumors.

**Table 2:** Follow up of patients

**No.**	**Age (years old)**	**Type**	**Surgical methods**	**Pathological type**	**Chemotherapy regimens**	**Follow-up**
1	36	Localized	Tumor cytoreductive surgery	Epithelioid type	No chemotherapy	Survived 109 m after surgery without tumor recurrence
2	47	Diffuse	Tumor cytoreductive surgery	Epithelioid type	(Pemetrexed + Cisplatin) *2	Died 21 m after surgery
3	56	Diffuse	Partial tumor resection	Epithelioid type	(Pemetrexed + Cisplatin) *3	Died 19 m after surgery
4	68	Diffuse	Partial tumor resection	Epithelioid type	(Pemetrexed + Cisplatin) *5	Died 33 m after surgery
5	67	Diffuse	Tumor cytoreductive surgery	Epithelioid type	(Pemetrexed + Cisplatin) *2	Died 17 m after surgery
6	70	Diffuse	Tumor cytoreductive surgery	Epithelioid type	Triplizumab *3	Lost to follow-up
7	69	Diffuse	Tumor cytoreductive surgery	Epithelioid type	(Pemetrexed + Cisplatin) *6	Survived, tumor recurrence
8	65	Diffuse	Partial tumor resection	Mixed	(Pemetrexed + Cisplatin) *4	Died 22 m after surgery
9	67	Diffuse	Partial tumor resection	Epithelioid type	(Pemetrexed + Cisplatin + Bevacizumab) *3	Survived, tumor recurrence
10	27	Localized	Tumor cytoreductive surgery	Epithelioid type	(Pemetrexed + Cisplatin + Bevacizumab) *2	Survived without tumor recurrence

*represents the number of chemotherapy treatments

#### Localized OMM

**Fig. 1:**
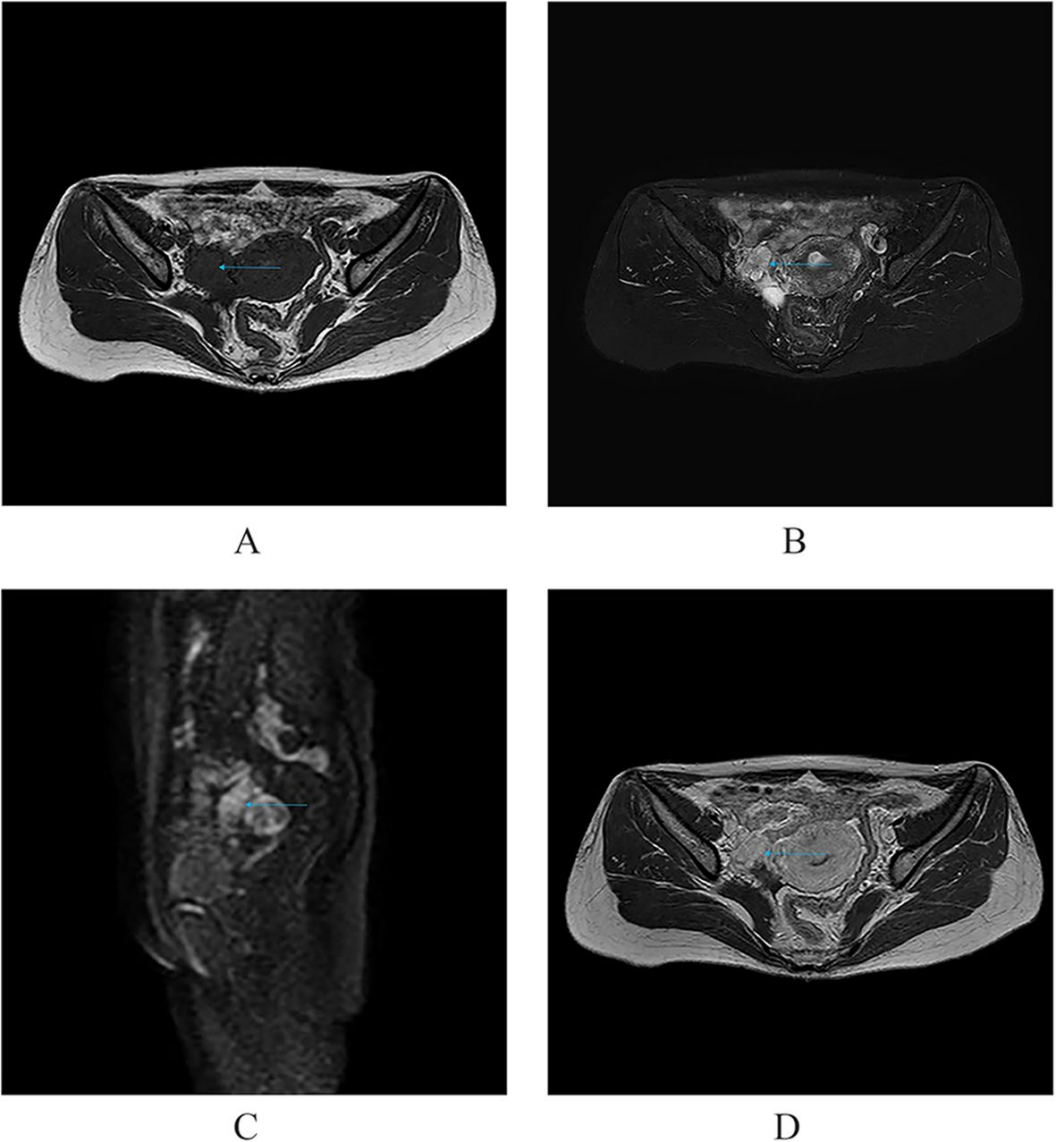
Limited OMM: Isointense on T1WI and muscular tissue, and markedly hyperintense on T2WI and DWI, which is significantly enhanced after enhancement. A linear “capsule” (long arrow) can be seen around it

Only enhanced MRI examination was performed in two patients with localized OMM, and they both developed unilateral disease, with mass sizes of 3.7*2.4*1.9 cm and 4.0*3.6*3.1 cm, respectively. The signal intensity of the tumor parenchyma on T1WI was equal to, or slightly lower than that of the muscle tissue and it was hyperintense on T2WI, with scattered and irregular hypointense signals visible therein and no liquefaction, partition, or mural nodule was seen (Fig. 1a-b). The DWI parenchyma was obviously hyperintense (Fig. 1c), and the tumor showed obvious heterogeneous enhancement after contrast administration (Fig. 1d). An incomplete semicircular “capsule” was observed around the tumor, which was hypointense on both T1WI and T2WI and showed no significant enhancement after enhancement (Fig. 1a,b,d).

#### Diffuse OMM

**Fig. 2:**
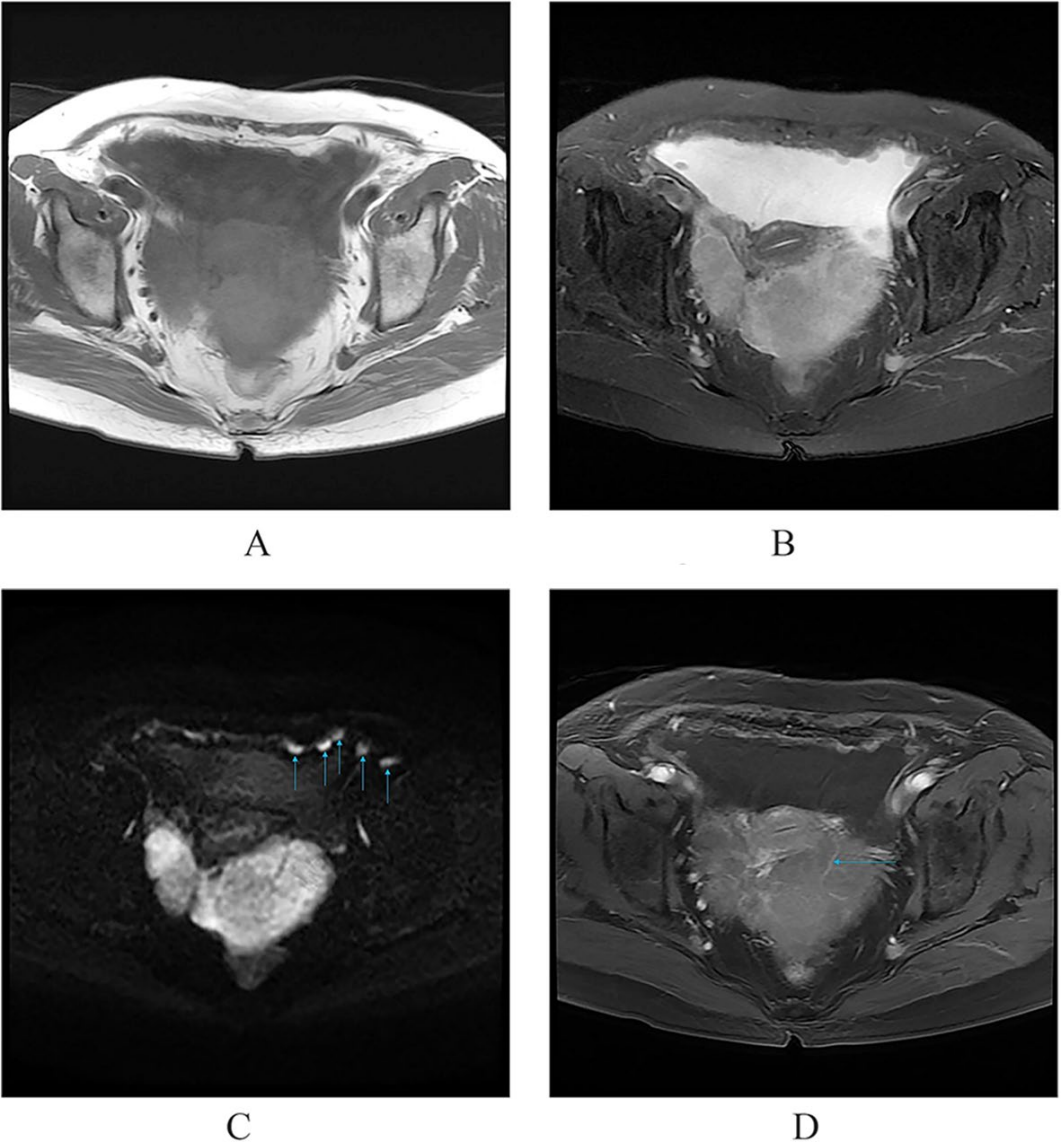
Diffuse OMM: huge tumor in the pelvis with unclear bilateral ovarian structures, dendritic vascular shadow within the tumor, regular course, and no truncation, dilation, and vascular lakes

**Fig. 3:**
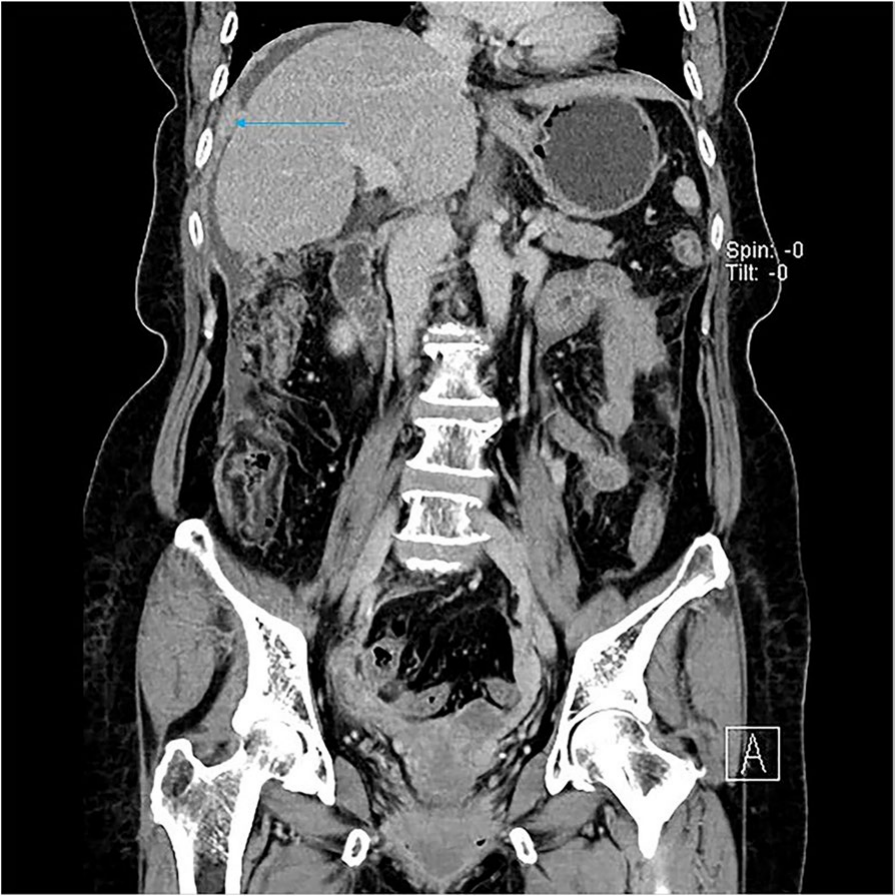
The subphrenic peritoneum shows wavy thickening (long arrow), with perihepatic ascites, a small amount of ascites in the pelvis, and no ascites in the hepatic and renal crypts

**Fig. 4:**
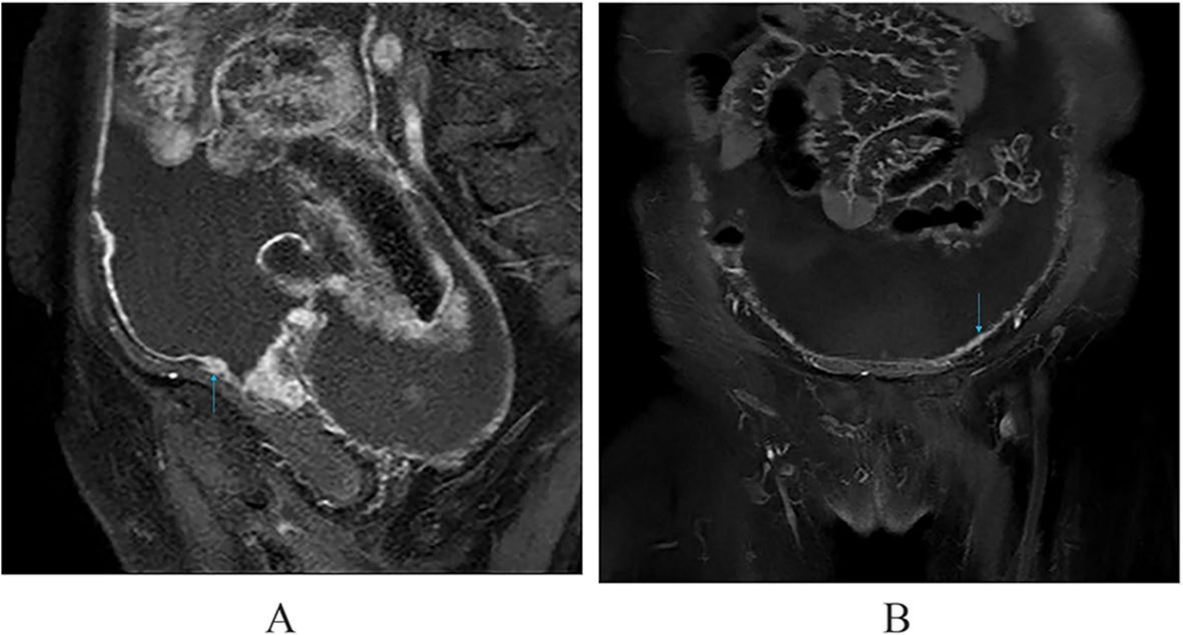
**a **Nodular or irregular thickening of the peritoneum. **b** Multiple small miliary nodules are attached to the surface

**Fig. 5:**
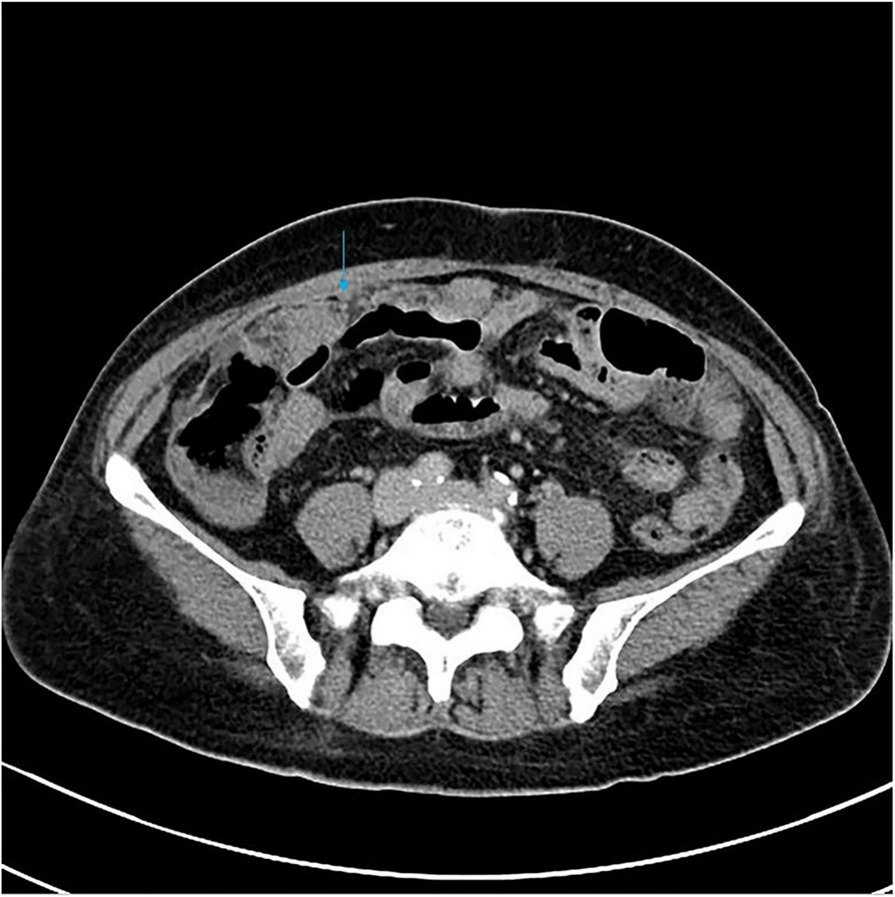
Nodular thickening of the parietal peritoneum

**Fig. 6:**
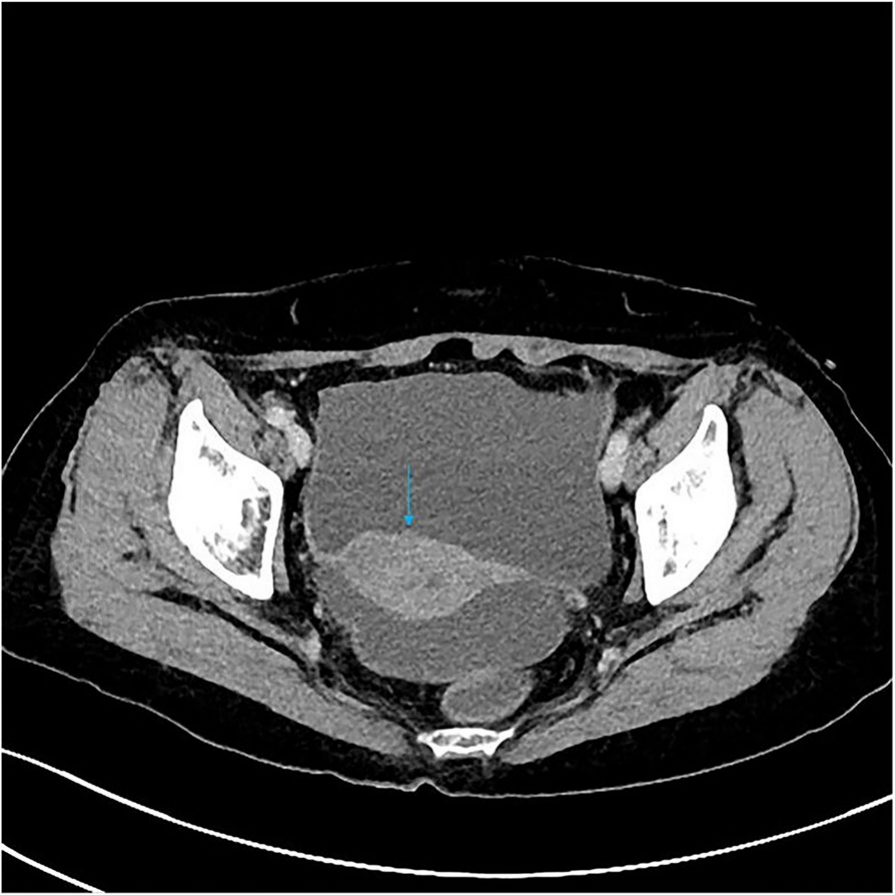
Nodular thickening of the peritoneum in the Douglas cul-de-sac

**Fig. 7:**
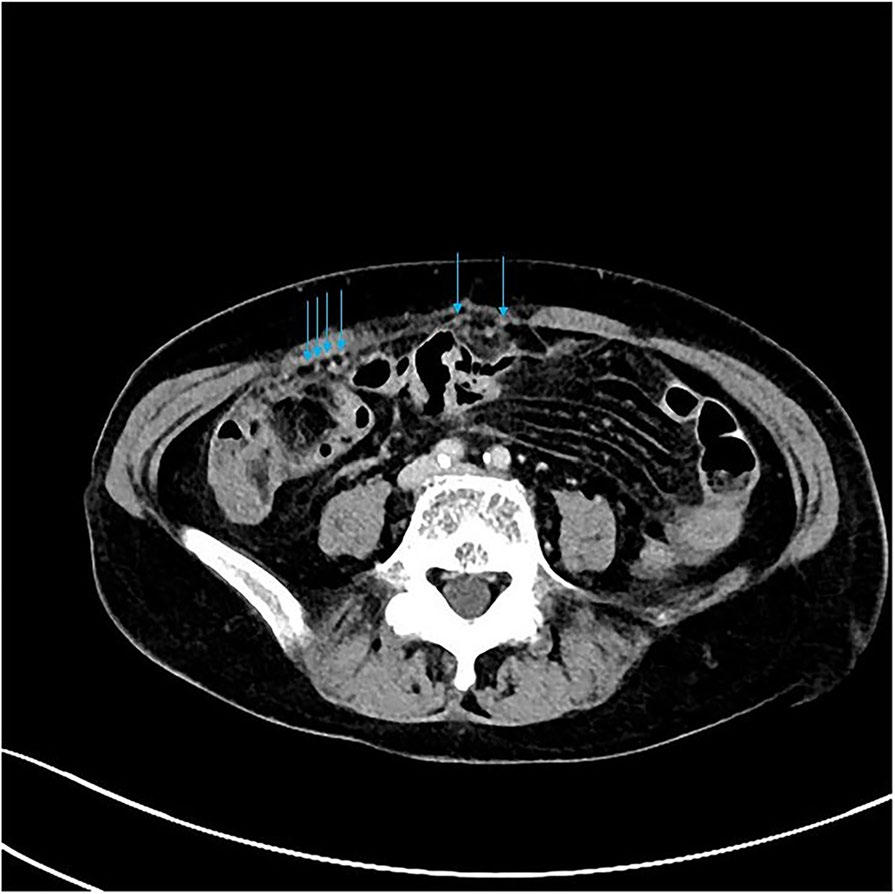
Scattered miliary nodules can be seen in the parietal peritoneum, with beaded distribution

**Fig. 8:**
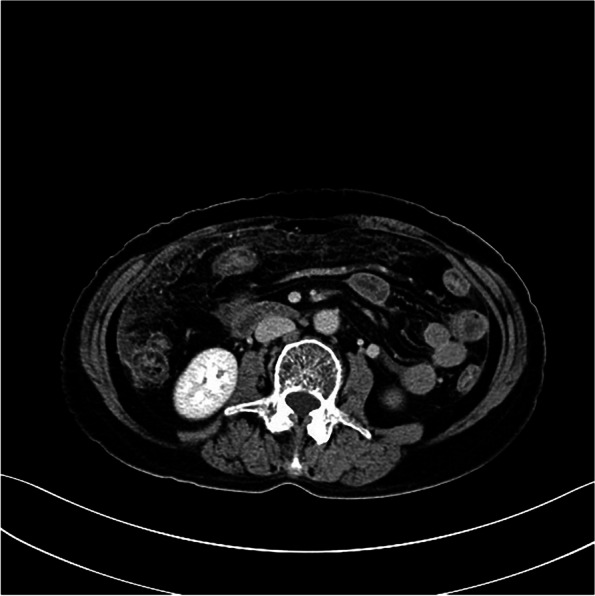
The greater omentum was cloudy, reticular thickened, and the omentum fused into a “cake” shape

**Fig. 9:**
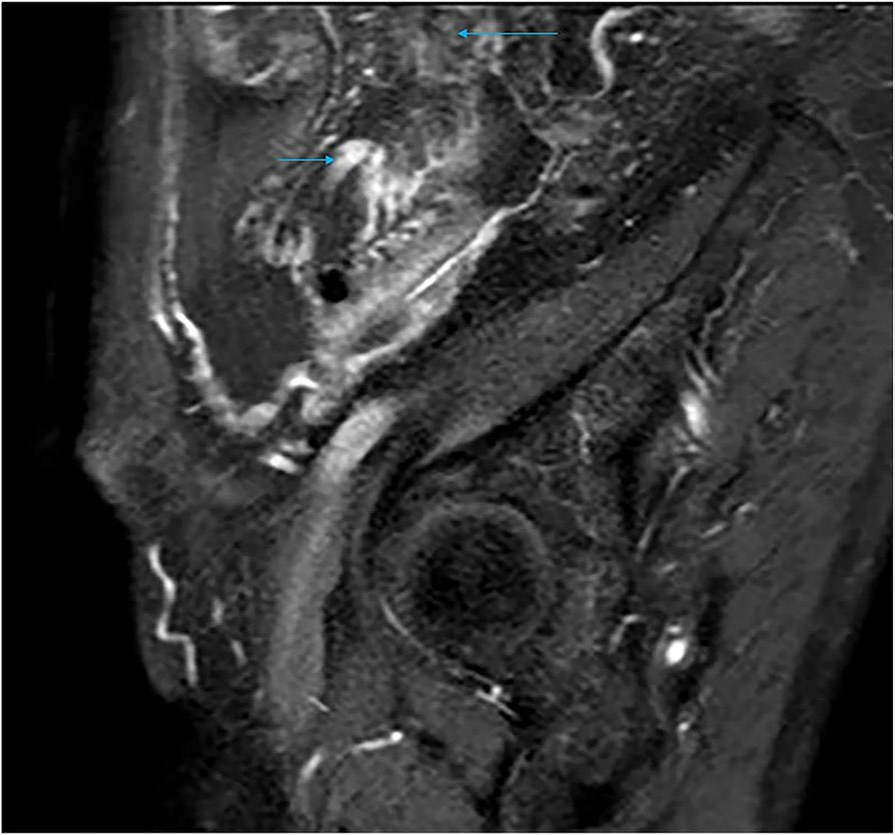
The omentum is turbid, the fat gap is unclear, and the omentum is reticular and thickened

**Fig. 10:**
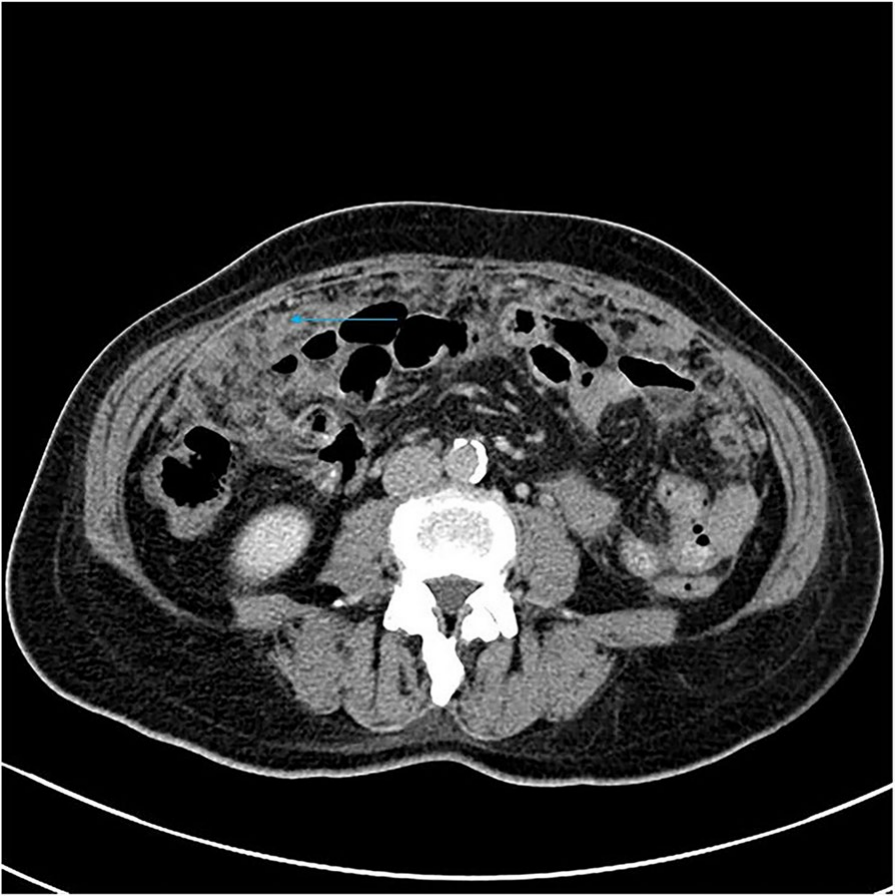
The greater omentum shows clusters of small nodules and “omental cake“

**Fig. 11:**
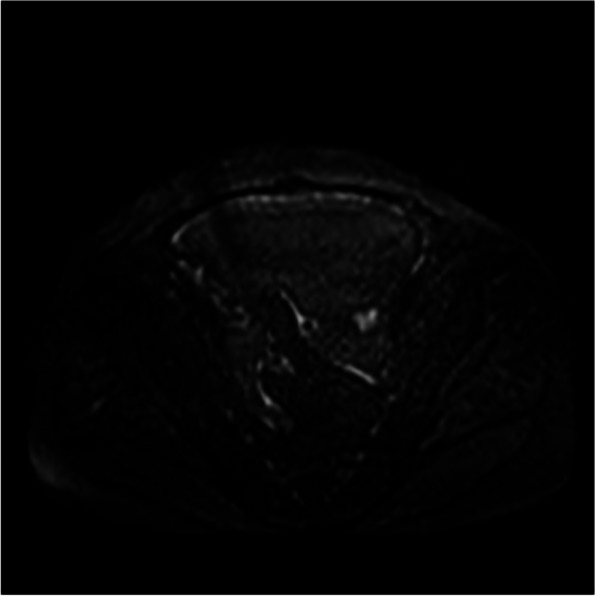
DWI tumor and peritoneum clearly shown against low signal

Among the eight patients with diffuse OMM, six underwent full abdominal contrast-enhanced CT and two underwent pelvic contrast-enhanced MRI. Six patients were presented with enlarged ovaries, of which, two had bilateral ovaries that were enlarged. Four had unilateral ovaries that were enlarged in spindle, irregular, or oval shapes, without clear demarcation from the tumor. One patient was presented with a huge pelvic mass with a diameter of 10 cm, which could not be distinguished from the ovarian structure. Dendritic vascular structure was observed inside, with no obvious truncation, distortion, dilation, stenosis, vascular lake, and stiffness of the blood vessels, or liquefaction, partition, and wall nodules (Fig. 2a-d). Eight patients had nodular, irregular, or wavy (Figs. 2c, 3, 4a and b, 5 and 6) thickening of the anterior abdominal wall peritoneum—four in the Douglas’ trap peritoneum and four in the subdiaphragmatic peritoneum, with scattered millet-like or hillock-like mural nodules on the surface (Figs. 4b and 7) that could be beaded (Figs. 2c and 7). The omentums of six patients were cloudy, the fat space was unclear, reticular thickening was observed, and multiple small nodules could be seen distributed in clusters or gathered into clusters (Figs. 8 and 9). The omentums of two patients were diffusely thickened and fused into a “cake-shape” (Figs. 8 and 10). The mass was isointense similar to the muscular tissue on T1WI, and obviously hyperintense on T2WI, without calcification, cystic degeneration, and liquefaction. The DWI tumor, diseased omentum, peritoneum, and superficial mural nodules showed obvious hyperintense signal, which was extremely clear against the surrounding hypointense tissue (Figs. 2c and 11). After enhancement, the tumor was moderately to markedly progressive (Figs. 2d and 4a).

All the eight patients had intraperitoneal ascites, including two cases with large ascites, four cases with medium volume ascites, and two cases with small volume ascites. Two patients had perihepatic ascites without hepatorenal crypt ascites while there was only a small amount of ascites in the pelvis. The density of ascites and the signals of each sequence were similar to those in urine, and there was no enhancement after enhancement. No swollen lymph nodes were found in the internal and external iliac arteries and common iliac artery group in all patients, and no metastasis was found in the abdominal parenchymal organs.

### Pathological examination results

**Fig. 12:**
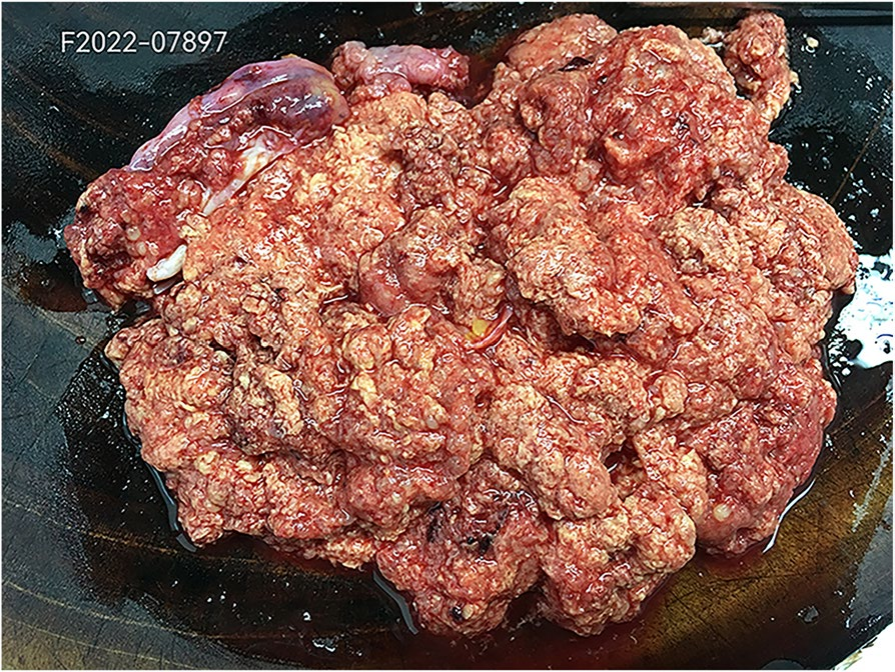
The right ovarian tumor is grayish yellow and grayish red, with soft texture and broken tissue

**Fig. 13:**
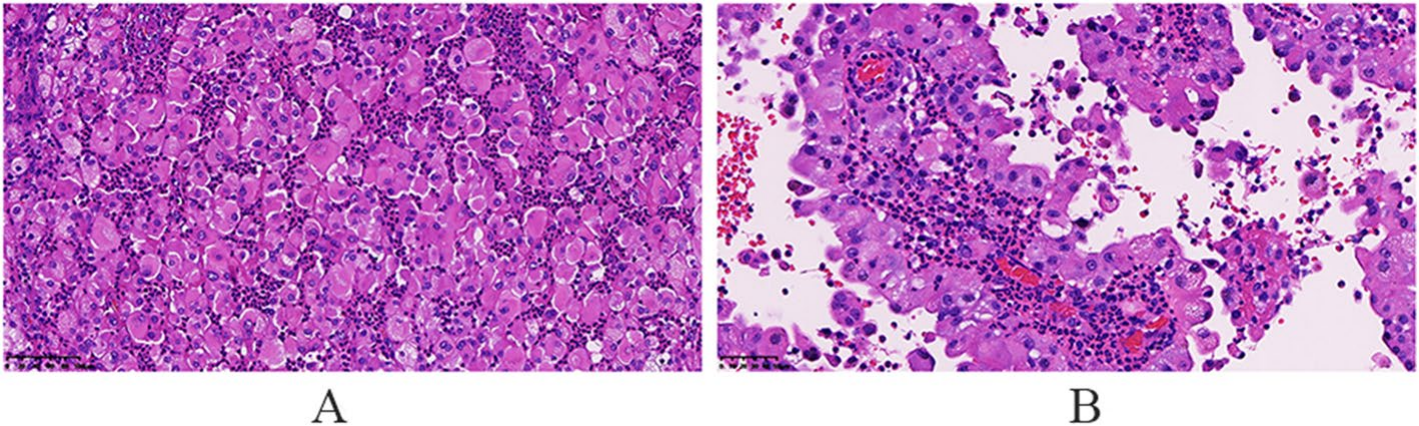
The tumor cells are epithelioid with abundant eosinophilic cytoplasm and clear cell membrane, and the tumor cells have a papillary structure

**Fig. 14:**
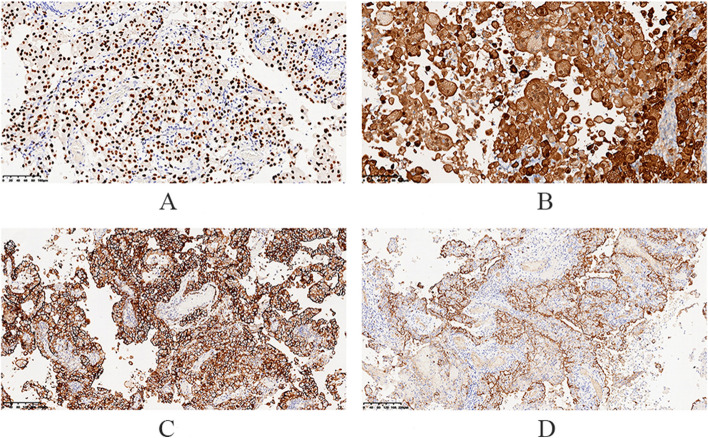
**A ** Tumor cell WT-1 pervaded positive. **B **Calretinin pervaded with strong positive. **C **D2-40 pervaded with strong positive. **D **MC(HBME1) positive

Some epithelial markers (CK(pan), CK7, EMA, and PAX8) were expressed in the tumor. Expressed mesothelial markers: Calretinin, D2-40, CK5/6, WT-1, MC (HBME1); Markers not expressed: EA(Ber-EP4), MOC31, CD15, ER, PR (Figs. 12, 13 and 14).

### Treatment and follow-up

All the 10 patients in this group underwent surgical treatment, and no severe complications occurred during the perioperative period. Two to six cycles of systemic chemotherapy using pemetrexed + cisplatin were performed in six patients postoperatively. Two patients received two and three cycles of pemetrexed + cisplatin + bevacizumab, respectively. One patient received three cycles of tripril monoclonal antibody immunotherapy and one patient did not receive chemotherapy because of no peripheral invasion and distant metastasis. Two patients with localized OMM survived, one for 109 months and one for 3 months, after operation. Among the eight patients with diffuse OMM, five died, and the postoperative survival time was 17 ~ 33 months, with the average survival time being 22.40 ± 6.23 months. Two patients survived for 15 months and 13 months after surgery, respectively, and one patient was lost to follow-up (Table 2).

## Discussion

### Clinical features

Malignant mesothelium (MM) is a malignant tumor originating from the mesothelium, [3, 4] and has an incidence rate of only 0.5 ~ 2/100,000. OMM is rare, accounting for about 0.03% of mesothelioma-related deaths [5]. The specific age group of high incidence of OMM is unknown and the majority of patients in this group are middle-aged and elderly patients with the average age being 57.2 ± 15.4 years. The proportion of patients over 47 years is 80%. The pathogenesis of this disease is unknown, and scholars believe that incidence of MM is related to exposure to asbestos [6]. Patients in China rarely have exposure to asbestos [7]. Only one out of 10 patients in this group had a history of asbestos exposure. The histopathological types of MM include epithelioid, sarcomatoid, and mixed types, with the epithelial type being more common [8]. The epithelial type accounted for 90% of the 10 patients in this group, which is consistent with literature. The disease is mostly asymptomatic in the early stage. When the tumor is significantly enlarged or metastasized, non-specific symptoms such as abdominal pain, abdominal distension, mass, and lower abdominal discomfort may appear. Since CA-125 widely exists in various mesothelial cell tissues, malignant mesothelioma CA-125 can also be elevated [9]. CA-125 in all the 10 patients in this group was increased slightly or moderately.

### Imaging signs

#### Localized OMM

Localized OMM is characterized by a tumor in the unilateral ovarian area, isointense signals similar to those in the muscle tissue on T1WI, and significantly higher signals than those in the muscle on T2WI and DWI. The substance of the OMM is obviously inhomogeneous enhanced after enhancement. No liquefaction, cystic degeneration, and partition are observed in the tumor. This serves as the primary point of differentiation from primary epithelial tumors of the ovary. An incomplete “capsule” is observed around the tumor, which is presumed to be the peritoneum around the tumor. The incomplete “capsule” may be caused by local invasion of the tumor and inflammatory adhesion.

#### Diffuse OMM

##### Ovarian manifestations

Diffuse OMM imaging is primarily not dominated by ovarian tumors, but shows ovarian enlargement, peritoneal and omental thickening with nodules and ascites, and other signs. In our case, diffuse OMM had mostly unremarkable ovarian enlargement, which was also one of the distinguishing points from other primary malignant ovarian tumors, such as epithelial tumors, germ cell tumors, and sex cord interstitial tumors. According to Hu et al., [10, 11] OMM should be defined as MM when all or most of the lesions are located in the ovary, while Fox and Wang et al., [11, 12] also suggest that enlargement of the ovary could be used to distinguish between primary and metastatic OMM. In our opinion, it is unscientific to assess where the primary lesion is located based on whether the ovary is enlarged or not. The mesothelial cells are mainly located on the ovarian surface [13] and the peritoneum is located around the ovary. When ovarian mesothelial cells turn into OMM, they can very easily invade the peritoneum. The peritoneum and omentum contain a large number of mesothelial cells, which provide good space for tumor spread and metastasis. They can rapidly develop in a short period of time, leading to a large number of peritoneal and omentum metastases, which present as diffuse OMM. Of course, tumors can also invade the ovary. A patient in our group had a large pelvic mass, which may suggest this growth pattern. In this patient, the intratumoral signal was relatively uniform, without liquefaction, partition, and mural nodules, and there was no significant deformation of the vascular structure, which could serve to distinguish it from malignant epithelial tumors of the ovary.

##### Peritoneal and omental manifestations and Ascites

In this study, the peritoneum was observed to be thick in an uneven cord-like or wavy shape, and mural nodules were seen on the surface of the peritoneum, which is consistent with the characteristics of implantable metastasis. The greater omentum was cloudy, and the fat gap was blurred or not visible, with scattered small nodules being visible. The omentum became reticular and thickened or fused into an “omental cake.“ No obvious mass-type lesion was observed in the abdominal cavity of our group. When the invasion of peritoneum and omentum was considered, the lesion was found to have mainly grown along the surface, and it was difficult for it to form a local mass. The tumors were isodense on CT scan, and gradually and obviously enhanced after enhancement. The signal intensity was isointense on T1WI and isointense on T2WI. DWI showed that the tumor tissue was the most sensitive. Against the background of low signal intensity, the tumor morphology showed streak-like, nodular, and pie-like hyperintense signals, and some beaded changes could be seen. MRI is obviously superior to CT in displaying the internal structures of the ovary and tumor as well as the invasion of peritoneum and omentum due to the characteristics of multi-parameter imaging. In particular, DWI sequences have a high degree of sensitivity, and accurate judgment can be made on the tumor involvement range without the use of contrast agents. However, due to the limitation of coils, the scope of MRI scan is limited to the pelvis, and the subdiaphragmatic lesions and upper abdominal ascites cannot be observed. For the same reason, the characteristics of tumor blood supply cannot be quantified. Therefore, when OMM is suspected, a combination of CT and MRI should be performed preoperatively.

Ascites could be seen in all the diffuse-type OMMs in this group. In the two patients who had perihepatic ascites, only a small amount of ascites was present in the pelvis and Douglas’ den, and the ascites was only located at the periphery of the liver, but there was no ascites in the hepatic and renal crypts, suggesting that the ascites originated from the diaphragmatic peritoneal lesions rather than the free pelvic ascites flowing to the abdominal cavity. This ascites distribution feature can also be used as a differentiating point between diffuse OMM and other primary ovarian malignant tumors. OMM can be transferred to the diaphragmatic peritoneum at an early stage, causing ascites, while other ovarian malignant tumors usually invade the peritoneum at the pelvic wall first, and then spread to the middle and upper abdomen later. The ascites in the abdominal cavity also shows characteristics of lower abdomen first and then upper abdomen.

Peritoneal thickening and ascites are also common in the differentiation of tuberculous peritonitis. The following signs may be helpful in differentiating the two: OMM-induced peritoneal thickening may be accompanied by mural nodules, whereas tuberculous peritonitis is dominated by smooth thickening; OMM often results in moderate to large amounts of ascites, while tuberculous peritonitis usually results in as few, or moderate amounts of ascites. Calcifications of tuberculous peritonitis and annular enhanced signs of enlarged lymph nodes are also rarely seen in OMM.

##### Lymph node manifestations

In epithelial ovarian tumors, lymph node metastasis occurs in 15%~30% of patients from the common iliac artery as well as internal and external iliac groups. No lymph node metastasis was found in 10 patients in this group. This is another differentiating point of OMM from other primary malignant tumors of the ovary.

### Differential diagnosis

#### Localized OMM is mainly differentiated from germ cell tumors and sex cord- stromal tumors

##### Germ cell tumors

The incidence group of localized OMM is mainly middle-aged and elderly women. There is no fat or bone components in the lesion, and no specific tumor markers. Patients with germ cell tumors are generally adolescents or women in their reproductive period. Teratomas can exhibit highly typical features such as mature fat and teeth. Dermoid cysts are mostly cystic components and may be accompanied by varying degrees of calcification. Endodermal sinus tumors occur at a younger age and can occur in children and women of childbearing age, with significant enhancement and may be accompanied by elevated AFP.

##### Sex cord stromal tumors

Both localized OMM and sex cord stromal tumors are presented as masses with clear boundary, with similar signals on T1WI. However, OMM shows high signal on T2WI and significantly uneven enhancement. However, due to the rich fibrous components, sex cord stromal tumors exhibit low or extremely low signal on T2WI and lack obvious enhancement, which is helpful in distinguishing them from OMM.

#### Diffuse OMM is mainly differentiated from malignant ovarian epithelial tumors and metastatic tumors

##### Malignant ovarian epithelial tumors

Diffuse OMM ovaries exhibit irregular enlargement without large masses and do not cause compression to surrounding organs. Malignant ovarian epithelial tumors can be seen as obvious masses, with a diameter of over 5 cm or even greater than 10 cm. They can also spread and grow into the abdominal cavity, compressing surrounding organs. OMM presents a solid structure with no internal separation, with equal or slightly low signal intensity on T1WI and uneven high signal intensity on T2WI. After enhancement, it mainly exhibits progressive moderate enhancement; Malignant ovarian epithelial tumors present as cystic or cystic solid structures, with uneven thickness of septa visible within the tumor. Solid components and septa show equal signal intensity on T1WI, while T2WI shows equal or slightly higher signal intensity. After enhancement, the cystic components can exhibit characteristic water like signals. In addition, due to the presence of a large number of mesothelial cells homologous to OMM in the peritoneum, OMM is more likely to have peritoneal dissemination as the main pathway of metastasis. Therefore, the scope and extent of peritoneal involvement are more obvious, and the “omental cake” area is more diffuse, while lymph node metastasis is a secondary pathway. No significant enlarged lymph nodes were found in this group of patients. Although malignant ovarian epithelial tumors can also undergo peritoneal metastasis, their main pathways of metastasis are direct invasion and lymphatic metastasis. Therefore, peritoneal and omental lesions are relatively mild, while surrounding lymph node enlargement is more common.

##### Metastatic tumors

The significance of medical history is crucial in distinguishing OMM from metastatic tumors. Ovarian metastatic tumors have a history of primary malignant tumors, with metastatic lesions mostly originating from organs such as the gastrointestinal tract, breast, and lungs. In terms of imaging findings, OMM mainly manifests as a slight enlargement of the ovary, rather than a significant mass, with moderate enhancement; Ovarian metastatic tumors can be seen with obvious masses, which are more common on both sides, and can be cystic, cystic solid, or solid masses. The enhancement mode is similar to that of the primary tumor.

### Treatment and follow-up

The treatment of OMM lacks authoritative guidance. Currently, with reference to the treatment scheme of peritoneal malignant mesothelioma, the combined therapy of cytoreductive surgery (CRS) with hyperthermal intra-peritoneal chemotherapy (HIPEC), systemic chemotherapy, intraperitoneal chemotherapy, immunotherapy, and molecular targeted therapy is mainly adopted [14, 15]. The survival time of OMM is unknown, while that of diffuse OMM reported by Clement et al., [16] is 18 ~ 44 months. In this group, the prognosis of the two patients with localized OMM was good, while that of the diffuse OMM was poor. The average survival time of the five dead patients with diffuse OMM was 22.40 ± 6.23 months after operation, one patient was lost to follow-up, and two patients were still alive 15 months and 13 months after operation, respectively. In summary, although OMM is rare, its imaging has certain characteristics. First, there is no cystic change, calcification, partition, or nodule on the inner wall of the tumor. Secondly, diffuse lesions do not mainly present with ovarian enlargement. Third, ascites can appear early in the upper abdomen. Last, but not least, the peritoneal mural nodules are also more representative. The shortcomings of this study are: First, due to the small number of cases, the diversity of tumor manifestations, and because all patients only underwent one examination of CT or MRI, one-sidedness in the evaluation of imaging manifestations is unavoidable. Second, the cases in this study originated from multiple centers and the different phases of MRI scan sequence and enhanced CT in each center may affect the comparison and observation of imaging signs.

## Data Availability

All data generated or analysed during this study are included in this article. Further enquiries can be directed to the corresponding author.

## References

[1] Boussios S, Moschetta M, Karathanasi A (2018). Malignant peritoneal Mesothelioma: clinical aspects, and therapeutic perspectives. Ann Gastroenterol.

[2] Zhang SL, Yu WY, Wang L (2017). A clinicopathological analysis of 4 cases with epithelioid malignant Mesothelioma of the ovary. J Clin Exp Pathol.

[3] Ohya M, Kobayashi M, Sozuki T (2019). Malignant peritoneal Mesothelioma diagnosed 50 years post–radiotherapy for Ovarian cancer in a patient with a history of multiple malignancies: an autopsy case. Mol Clin Oncol.

[4] Kapoor R, Kuttikat PG, Vaiphei K (2015). A case report of peritoneal malignant Mesothelioma presenting as primary ovarian mass. J Cancer Res Ther.

[5] Merino MJ (2010). Malignant Mesothelioma mimicking Ovarian cancer. Int J Surg Pathol.

[6] Arif Q, Husain AN (2015). Malignant Mesothelioma diagnosis. Arch Pathol Lab Med.

[7] Guo XX, Li YC (2010). Analysis of 16 patients with peritoneal malignant Mesothelioma misdiagnosed as ovarian carcinoma. J Practical Obstet Gynecol.

[8] An YX. Wei LX.Malignant Mesothelioma: clinicopathological analyses of 100 cases. J Diag Pathol,2015(3):156–158. 10.3969/j.issn.1007-8096.2015.03.008.

[9] Zhang J (2003). Peritoneal malignant Mesothelioma misdiagnosed as Ovarian cancer: one case report. J Radioimmunol.

[10] Hu LN, Li L, Wang XQ (2015). Ovarian malignant Mesothelioma of both sides:a clinicopatho- logical analysis. J Diag Pathol.

[11] Wang M, Ma S, Zhao Z (2019). A case report of ovarian epithelioid malignant mesothelioma and literature review. J Int Reprod Health/Fam Plan.

[12] Fox H (1993). Primary neoplasia of the female peritoneum. Histopathol.

[13] Yang Y, Liu T, Li G (2013). MSCT presentation of diffuse malignant peritoneal Mesothelioma in women. Chin Imaging J Integr Traditional Western Med.

[14] The Chicago Consensus on peritoneal surface malignancies. : Management of peritoneal Mesothelioma. Ann Surg Oncol. 2020,27(6):1774–9. 10.1245/s10434-020-08324-w. 10.1245/s10434-020-08324-w 32285273

[15] Xu XH, Chen YC, Xu YL, Feng ZL, Liu QY, Guo X (2021). Garcinone E blocks autophagy through lysosomal functional destruction in Ovarian cancer cells. World J Tradit Chin Med.

[16] Clement PB, Young RH, Scully RE (1996). Malignant mesotheliomas presenting as ovarian masses: a report of nine cases, including two primary ovarian mesotheliomas. Am J Surg Pathol.

